# Automated diagnosis and staging of Fuchs’ endothelial cell corneal dystrophy using deep learning

**DOI:** 10.1186/s40662-020-00209-z

**Published:** 2020-09-01

**Authors:** Taher Eleiwa, Amr Elsawy, Eyüp Özcan, Mohamed Abou Shousha

**Affiliations:** 1 Bascom Palmer Eye Institute, Miller School of Medicine, University of Miami, Miami, Florida 33136 USA; 2 Department of Ophthalmology, Faculty of Medicine, Benha University, Benha, Egypt; 3 Electrical and Computer Engineering, University of Miami, Coral Gables, Florida USA; 4 Net Eye Medical Center, Gaziantep, Turkey; 5 Biomedical Engineering, University of Miami, Coral Gables, Florida USA

**Keywords:** Optical coherence tomography, Descemet’s membrane, Corneal endothelium, Fuchs endothelial dystrophy

## Abstract

**Background:**

To describe the diagnostic performance of a deep learning algorithm in discriminating early-stage Fuchs’ endothelial corneal dystrophy (FECD) without clinically evident corneal edema from healthy and late-stage FECD eyes using high-definition optical coherence tomography (HD-OCT).

**Methods:**

In this observational case-control study, 104 eyes (53 FECD eyes and 51 healthy controls) received HD-OCT imaging (Envisu R2210, Bioptigen, Buffalo Grove, IL, USA) using a 6 mm radial scan pattern centered on the corneal vertex. FECD was clinically categorized into early (without corneal edema) and late-stage (with corneal edema). A total of 18,720 anterior segment optical coherence tomography (AS-OCT) images (9180 healthy; 5400 early-stage FECD; 4140 late-stage FECD) of 104 eyes (81 patients) were used to develop and validate a deep learning classification network to differentiate early-stage FECD eyes from healthy eyes and those with clinical edema. Using 5-fold cross-validation on the dataset containing 11,340 OCT images (63 eyes), the network was trained with 80% of these images (3420 healthy; 3060 early-stage FECD; 2700 late-stage FECD), then tested with 20% (720 healthy; 720 early-stage FECD; 720 late-stage FECD). Thereafter, a final model was trained with the entire dataset consisting the 11,340 images and validated with a remaining 7380 images of unseen AS-OCT scans of 41 eyes (5040 healthy; 1620 early-stage FECD 720 late-stage FECD). Visualization of learned features was done, and area under curve (AUC), specificity, and sensitivity of the prediction outputs for healthy, early and late-stage FECD were computed.

**Results:**

The final model achieved an AUC of 0.997 ± 0.005 with 91% sensitivity and 97% specificity in detecting early-FECD; an AUC of 0.974 ± 0.005 with a specificity of 92% and a sensitivity up to 100% in detecting late-stage FECD; and an AUC of 0.998 ± 0.001 with a specificity 98% and a sensitivity of 99% in discriminating healthy corneas from all FECD.

**Conclusion:**

Deep learning algorithm is an accurate autonomous novel diagnostic tool of FECD with very high sensitivity and specificity that can be used to grade FECD severity with high accuracy.

## Background

Fuchs’ endothelial corneal dystrophy (FECD) is one of the leading indications of keratoplasty, with a 4% prevalence in those above the age of 40 years old in the US [1–5]. FECD is a bilateral asymmetric disease of the corneal endothelium characterized by progressive endothelial cell loss with formation of excrescences known as guttae that may result in corneal decompensation and decreased vision [6]. The tremendous development in the surgical and non-surgical therapeutics for FECD makes the early diagnosis of FECD before the development of irreversible microstructural changes crucial for obtaining best visual results [7–15].

FECD can be diagnosed using slit lamp examination, specular microscopy, corneal thickness measurement, and confocal microscopy. However, these tools are incapable of monitoring the chronological changes of the disease or predicting its progression, especially after cataract extraction. Although clinical diagnosis using slit lamp examination may be the gold standard, slit lamp examination does not account for the presence of subclinical edema, and isolated measurement of central corneal thickness is not always representative of corneal edema [16, 17]. Regarding specular and confocal microscopy, regional differences between guttate areas and visible cells, and potential sampling errors with limited field of view render the corneal measurements imprecise [18, 19]. Recently, pachymetry maps and posterior corneal curvature patterns generated with Scheimpflug tomography have been reported to facilitate the identification of subclinical edema in cases with FECD [4]. However, at least 1 of the tomographic features of interest was present in 7% of control eyes and 42% of the FECD cases with no edema. Thus, it is important to consider coexisting subtle corneal pathologies prior to interpreting the tomographic maps [4, 5]. Currently, with the advent of anterior segment optical coherence tomography (AS-OCT), it has become credible to perform non-contact in vivo imaging to evaluate the corneal microstructure of FECD cases at a quasi-histologic level [20–23]. Nevertheless, there is still a barrier in deploying this technology for clinical practice due to the lack of automated reliable and accurate analysis of OCT scans [24].

Several studies have demonstrated the utility of deep learning in the field of ophthalmology [25–31]. Deep learning enables computers to execute direct classification from images rather than through features recognition prespecified by human experts. This is accomplished by training algorithmic models on images with accompanying labels (e.g., OCT images categorized manually for the presence or absence of FECD), such that these models can be used to classify new images with similar labels. The models are neural networks that are constructed of an input layer (which receives, for example, the OCT image), followed by multiple layers of nonlinear transformations to produce an output (e.g., FECD present or absent) [28]. So far, few studies have focused specifically on automated detection of corneal disease using AS-OCT compared to retinal diseases and glaucoma [32]. To the best of our knowledge, there is no report on the use of deep learning for diagnosing FECD. However, automated detection and staging of FECD might be very useful to better counsel patients about their disease and the available treatment options (e.g., FECD screening before cataract surgery). Therefore, the primary aim of this study was to assess the performance of a deep learning algorithm not only for the detection of FECD, but also to identify early-stage disease without clinically evident edema using OCT images.

## Materials and methods

### Study design and participants

This study was approved by the University of Miami Institutional Review Board (ID 20180699). The study design complied with the Health Insurance Portability and Accountability Act (HIPAA), and the research was conducted in accordance with the tenets of the Declaration of Helsinki. All subjects provided written informed consent before participation.

A total of 18,720 AS-OCT images (9180 healthy; 5400 early-stage FECD; 4140 late-stage FECD) of 104 eyes (81 patients) were collected. Patients were prospectively and consecutively recruited from June 2018 to September 2019 at the Bascom Palmer Eye Institute. Images were used to train and test a deep learning algorithm to automatically diagnose and grade the severity of FECD in AS-OCT images. FECD was diagnosed clinically by the presence of guttae, with or without clinically evident edema. Eyes were either phakic or pseudophakic with an endocapsular posterior chamber intraocular lens implant in the studied groups, without any history of uveitis.

Subjects were excluded from the study if they had inflammatory ocular diseases, ocular surface diseases, glaucoma and systemic diseases with ocular involvement. Patients with history of ocular surgery (except uneventful cataract extraction with endocapsular intraocular lens implantation at least 6 months prior to enrollment), contact lens wear, or using topical (except artificial tears) or systemic medications that could affect the cornea were excluded. Slit lamp examination was done on each eye by a masked cornea specialist in order to assign the examined cornea into either a healthy cornea or FECD group. Furthermore, FECD eyes were clinically graded according to the following guidelines: grade 1: non-confluent guttae; grade 2: presence of any area of confluent guttae, but without clinical edema; grade 3: confluent guttae with clinical edema; grade 4: edema associated with whitening or haze [33]. We grouped grade 1 and 2 into early-stage FECD, and grade 3 and 4 into late-stage FECD [19, 23].

### Test methods

**Fig. 1:**
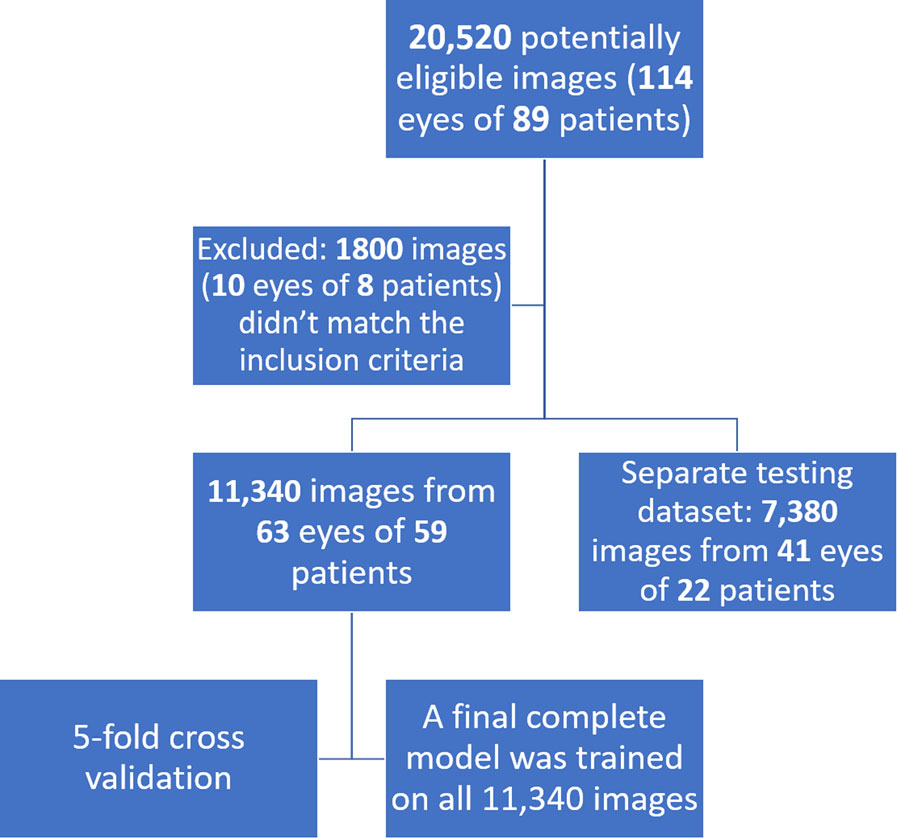
Flow-chart illustrating the number of anterior segment optical coherence tomography (AS-OCT) images used to develop, train and test the deep learning algorithm

Anterior segment high-definition optical coherence tomography (HD-OCT; Envisu R2210, Bioptigen, Buffalo Grove, IL, USA) was performed for each participant, using a 6 mm radial scan pattern centered on the corneal vertex with 180 cross-sectional images. Each participant was asked to look at a central fixation target; the presence of a visible specular reflection in all images of the scan confirmed an optimal centration [34, 35]. Then, labels including names, diagnoses, age, and sex were linked to the AS-OCT corneal images. The patients were organized by clinical category; healthy, early-stage, and late-stage FECD. For the training, validation, and testing procedures, all images were anonymized. A total of 20,520 AS-OCT images were captured from 114 eyes of 89 patients throughout the study. After quality check and reviewing the patients’ medical records and sorting the eyes into the 3 different groups based on the patient selection criteria stated above, 1800 images from 10 eyes of 8 subjects were excluded, and 18,720 images from 104 eyes (81 patients) were selected to develop and validate the model (Fig. 1). Quality check was performed manually by 4 trained independent operators by removing images that contained any of the following: decentralization, blinking, missing parts of the endothelium or epithelial layers of the cornea, and poor signal to noise ratio.

To simplify the creation of a deep learning model, the deep learning model was based on visual geometry group with 19 layers (VGG19) and transfer learning, with parameters of the model pre-trained on ImageNet dataset, using MATLAB 2019b (MathWorks, Natick, MA) [36, 37]. We tested the algorithm using 5-fold cross-validation on the dataset containing 11,340 images, maintaining the proportion of samples of each class per fold. This testing process trained 5 distinct algorithms with 9180 (80%) of these images (3420 healthy; 3060 early-stage FECD 2700 late-stage FECD), each holding off a discrete validation block of 2160 (20%) images (720 healthy; 720 early-stage FECD; 720 late-stage FECD) [32, 38, 39]. Mean parameters were calculated from 5 test runs on the corresponding held-off data by comparing the model’s predictions against the ground truth as determined by cornea specialists. The assignment toward the training and testing set was performed randomly. Lastly, a final complete model was trained on all 11,340 images before testing on 7380 of the above-mentioned but not seen AS-OCT scans of 41 eyes captured using the same machine (5040 healthy; 1620 early-stage FECD; 720 late-stage FECD). The deep learning model automatically computed the prediction output for each class between 0 and 1.

To visualize the learned patterns by the network, we plotted the activations values for different convolutional layers, and performed an occlusion test by repeatedly replacing patches in an image with random values to compute the probability of identifying the disease by our network [40].

### Statistical analysis

Statistical analyses were performed using SPSS software version 26.0 (SPSS, Chicago, IL, USA) and MATLAB 2019b (MathWorks, Natick, MA). Continuous data were summarized with means and standard deviations while dichotomous data were summarized with proportions. Comparisons between groups were performed using Generalized Estimating Equations (GEE) methods to account for the correlation between two eyes of the same patient [41]. Residuals of the fitted models were examined to assess model performance and Box-Cox methods were used to identify appropriate transformations to effect normality for significance testing when necessary [42]. We generated area under the receiver operating characteristic curve (AUC) as a metric to measure the accuracy of our model, reporting related sensitivity and specificity parameters.

## Results

### Participants

Our study included 104 eyes from 81 participants; the breakdown included 53 eyes of 46 FECD patients, and 51 eyes of 35 healthy subjects of similar age and sex (Table 1).

**Table 1:** Characteristics of study groups

		Healthy Group (51 eyes of 35 patients)	FECD Group		***p*** value
			Early-stage (30 eyes of 26 patients)	Late-stage (23 eyes of 20 patients)	
FECD clinical grade^9^ Grade: N (%) of eyes		–	1: 14 (26%), 2: 16 (33%)	3: 11 (17%), 4: 12 (22%)	
Number of eyes	Phakic eyes	31 (60%)	27 (51%)		*P* = 0.449
	Pseudophakic eyes (PC.IOL)	20 (40%)	26 (49%)		
Sex	Female	24 (47%)	33 (62%)		*P* = 0.078^*^
	Male	27 (53%)	20 (38%)		
Age (range) years		65 (50-82)	69 (50-95)		*P* = 0.118^**^
Mean healthy cornea prediction output	Image level	0.99 ± 0.001	0.09 ± 0.012	0.00 ± 0.00	***P <*** 0.001^**^
	Eye level	0.995 ± 0.003	0.018 ± 0.008	0.00 ± 0.00	***P <*** 0.001^**^
Mean early-stage FECD prediction output	Image level	0.004 ± 0.001	0.755 ± 0.018	0.205 ± 0.04	***P <*** 0.001^**^
	Eye level	0.002 ± 0.001	0.855 ± 0.083	0.033 ± 0.028	***P <*** 0.001^**^
Mean late-stage FECD prediction output	Image level	0.001 ± 0.00	0.145 ± 0.015	0.795 ± 0.043	***P <*** 0.001^**^
	Eye level	0.0001 ± 0.00	0.155 ± 0.087	0.967 ± 0.028	***P <*** 0.001^**^

*FECD* Fuchs’ endothelial cell corneal dystrophy, *PC.IOL* posterior chamber intraocular lens

95% CI: 95 percent confidence interval on the difference between groups

Values are presented as median (range) for age

*P value was calculated using generalized estimating equations (GEE) with logistic link function and exchangeable correlation matrix

**P value was calculated using GEE with identity link function and exchangeable correlation matrix

### Training, validation and testing procedures

**Fig. 2:**
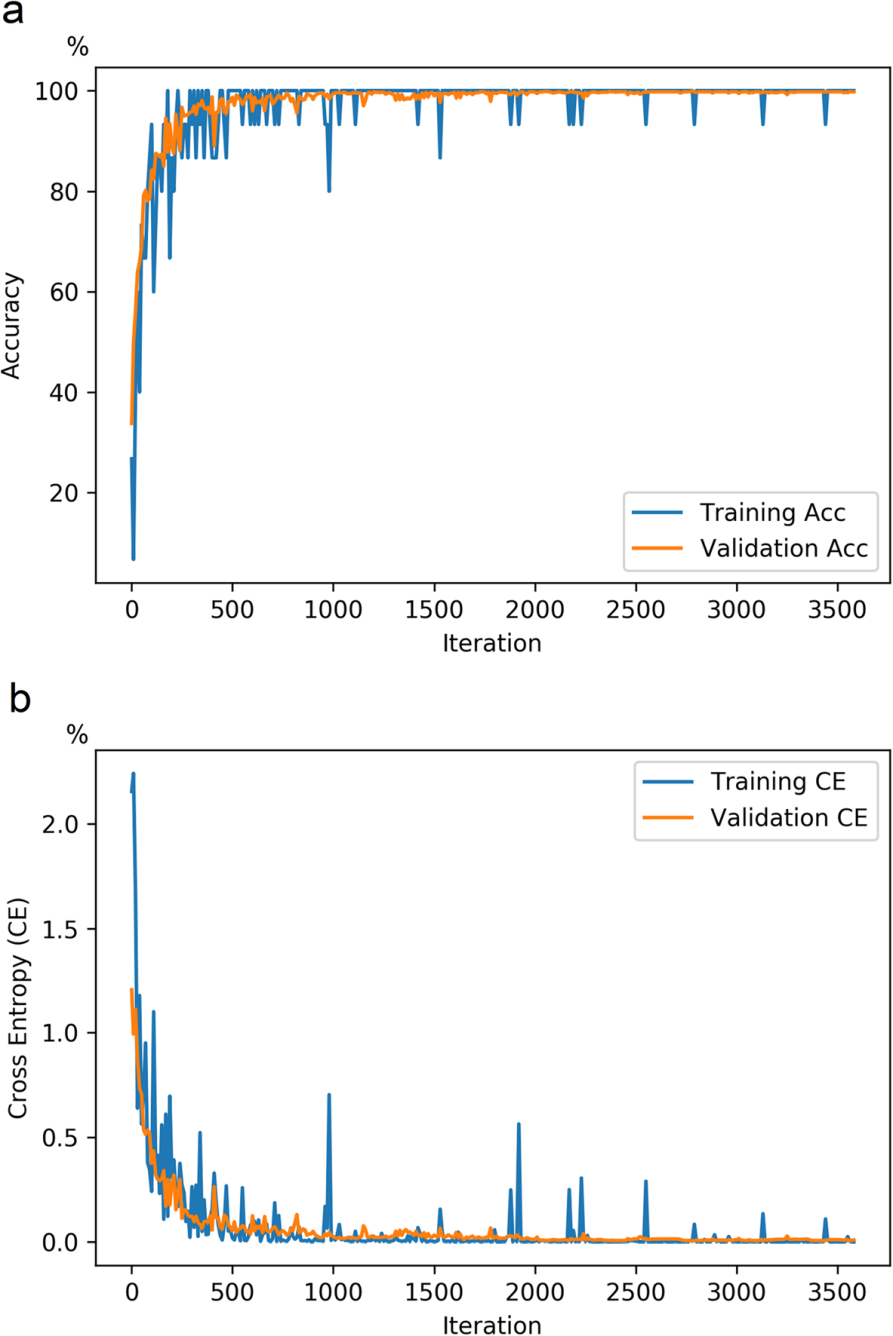
Line charts showing the model’s performance [in percentage] during the training procedure. **a** line chart showing continuous increase in both training accuracy and validation accuracy, and **b** line chart showing that cross entropy demonstrated a continuous decline to a final value less than 0.01

**Fig. 3:**
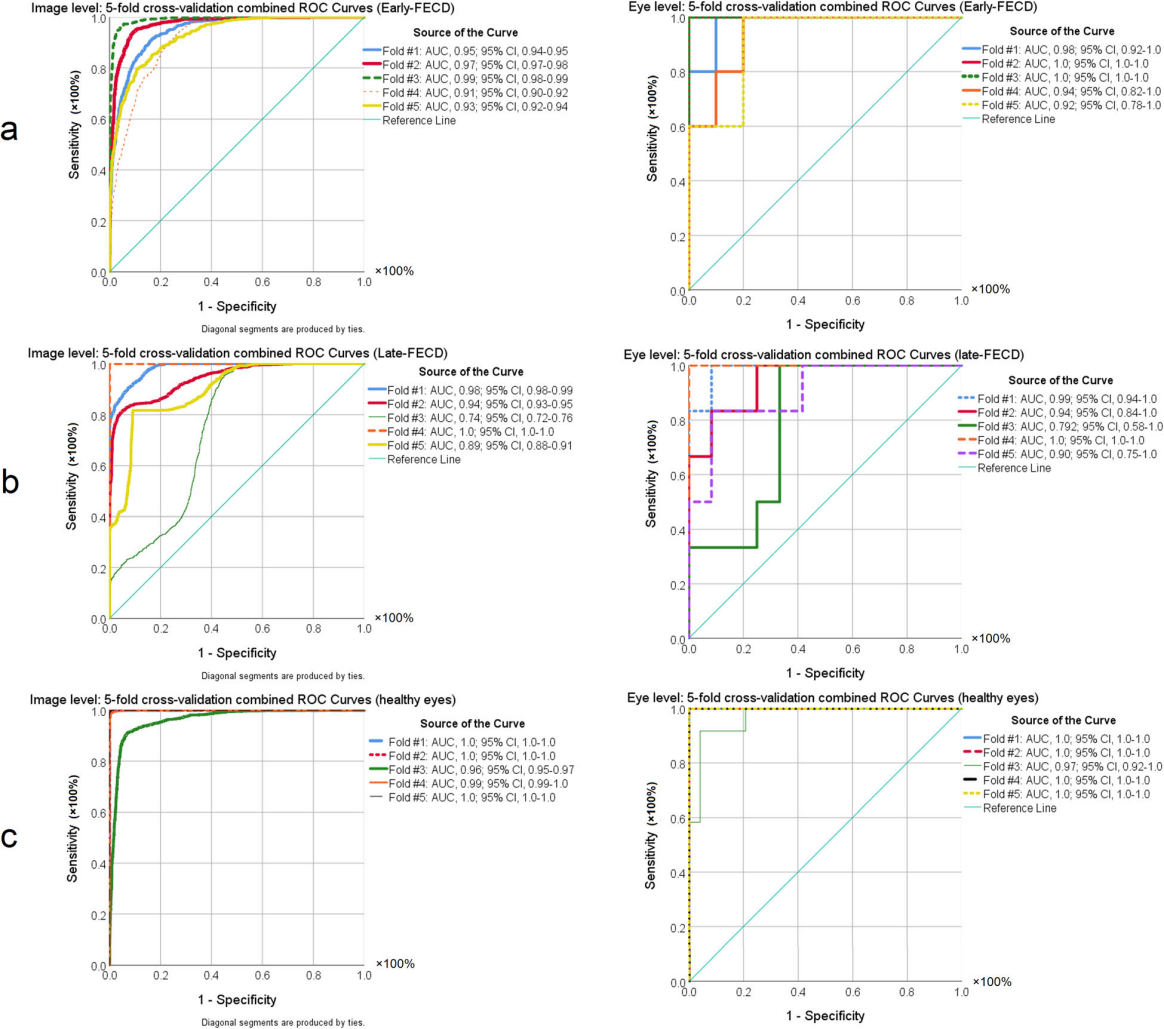
Receiver operating characteristic (ROC) curves of the five-fold cross-validation for the model based on the image- (left column) and eye-level (right column) classification accuracy for (**a**) early- and (**b**) late-stage Fuchs endothelial cell dystrophy (FECD) and (**c**) healthy eyes

After completing the training procedure, both training accuracy and validation accuracy demonstrated a continuous increase and were greater than 99%. In agreement with improvement of the model’s performance during the training process, cross entropy showed a continuous decline to a final value less than 0.01 (Fig. 2). Figure 3 demonstrates the receiver operating characteristic curves of the five-fold cross-validation for the model based on the image and eye-level classification accuracy. During cross-validation, the algorithm scored an average area under the receiver operating characteristic curve (AUC) of 0.95 (95% CI, 0.91–0.99), with a 91% (95% CI, 85–98%) sensitivity and an 85% (95% CI, 77–94%) specificity for identifying early FECD.

Using 7380 unseen AS-OCT images from 41 eyes, a highly significant difference was found between the mean prediction outputs for each class (Table 1). The mean early-FECD prediction output was 0.86 ± 0.27 in the early-stage FECD group as compared to 0.03 ± 0.04 in the late-stage FECD group and 0.01 ± 0.005 in the group with healthy corneas (*P* < 0.001). The mean late-FECD prediction output was 0.97 ± 0.04 in the late-stage FECD group while it was 0.16 ± 0.29 in the early-stage FECD group and 0.001 ± 0.0003 in the group with healthy corneas (*P* < 0.001). The mean healthy prediction output was 0.99 ± 0.02 in the healthy corneas versus 0.00 in the late-stage FECD group and 0.02 ± 0.03 in the group with early-stage FECD group (*P* < 0.001).

**Fig. 4:**
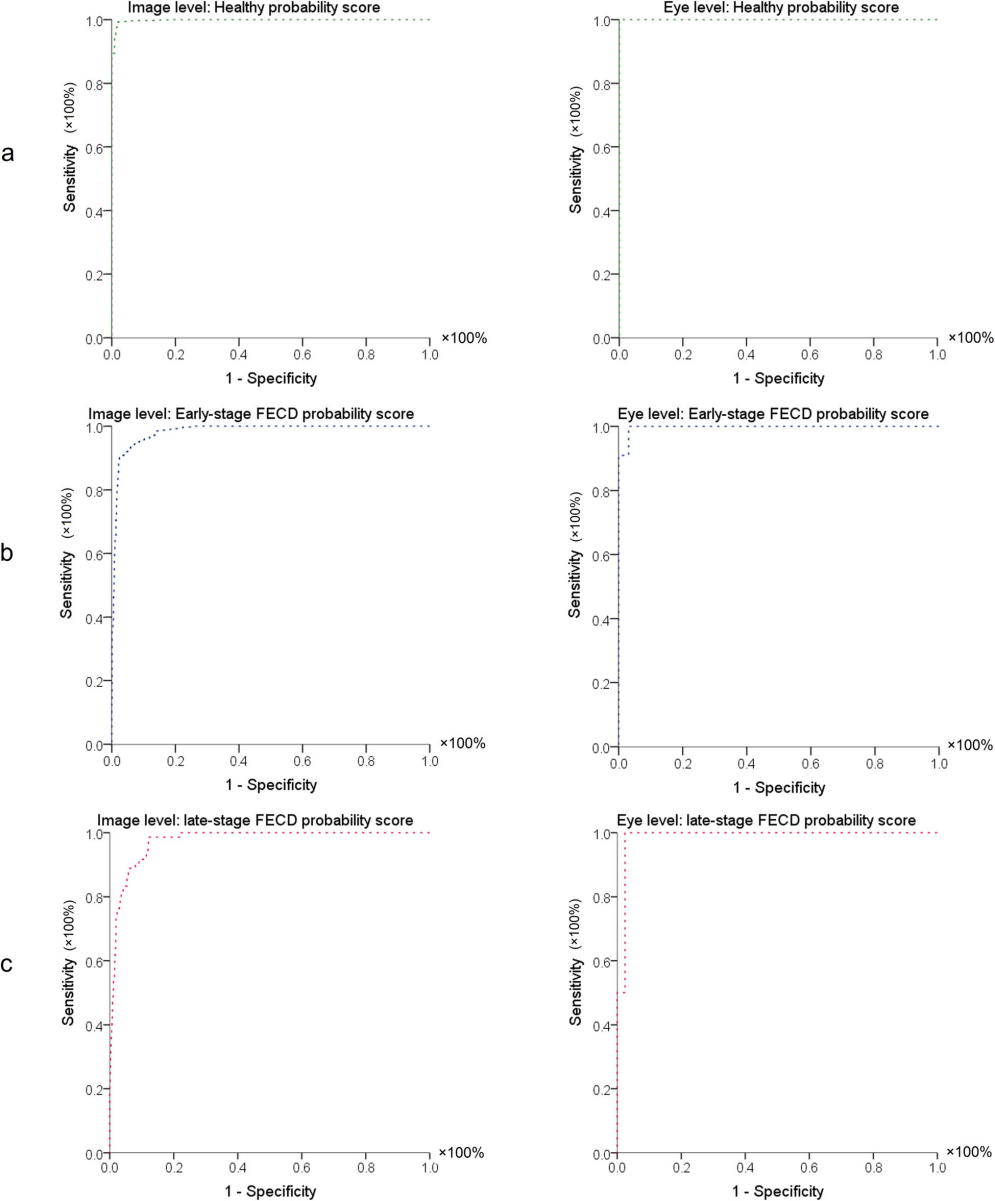
Receiver operating characteristics (ROC) graphs of (**a**) healthy cornea prediction output, (**b**) early-stage Fuchs’ endothelial corneal dystrophy (FECD) prediction output, and (**c**) late-stage FECD prediction output in the corresponding class at both the image level (left column) and eye level (right column)

Table 2 summarizes the diagnostic performance of early-stage FECD prediction output, late-stage FECD prediction output, and healthy cornea prediction output at both image and eye levels. At the image level, 0.3% was misclassified as early-stage FECD. While in early-stage FECD, 6% was misclassified as healthy, and 13% as late-stage FECD. From late-stage FECD eyes, 14% was diagnosed as early-stage FECD. Nevertheless, per eye level, only 9% early stage FECD was misclassified as late FECD. For identifying early stage FECD at image level, early-stage FECD prediction output had AUC of 0.984 ± 0.003 with a specificity of ≥97% and sensitivity up to 100%. Regarding late-stage FECD at image level, late-stage FECD prediction output achieved AUC of 0.974 ± 0.005 with a specificity of 92% and a sensitivity up to 100%. For discriminating healthy corneas from early and late-stage FECD, healthy cornea prediction output had an AUC of 0.998 ± 0.001 with a specificity 98% and a sensitivity of 99%. Per eye level, all the probability scores achieved 100% sensitivities with a specificity ≥97% (Table 2 and Fig. 4).

**Table 2:** Final model sensitivity, specificity, and area under the receiver operating characteristic curve measures of prediction outputs of early-stage FECD, late-stage FECD, and healthy cornea at both image and eye levels. All AUC *P*-values < 0.001 for all parameters

	**Image level (*n*** **= 7380 images)**		
	**Healthy cornea prediction output**	**Early-stage FECD prediction output**	**Late-stage FECD prediction output**
AUC ± SE (95% CI)	0.998 ± 0.001 (0.997, 0.999)	0.984 ± 0.003 (0.979, 0.999)	0.974 ± 0.005 (0.964, 0.985)
Sensitivity	99%	91%	92%
Specificity	98%	97%	91%
Cutoff value	0.93	0.04	0.01
	**Eye level (*n*** **= 41 eyes)**		
	**Healthy cornea prediction output**	**Early-stage FECD prediction output**	**Late-stage FECD prediction output**
AUC ± SE (95% CI)	1.0 (1, 1)	0.997 ± 0.005 (0.988, 1.0)	0.988 ± 0.017 (0.954, 1.0)
Sensitivity	100%	100%	100%
Specificity	100%	97%	98%
Cutoff value	0.49	0.03	0.67

• *FECD =* Fuchs’ endothelial cell corneal dystrophy; *AUC =* Area under the curve; *SE =* Standard error; 95% CI: 95% confidence interval on the difference between groups. Specificity, sensitivity, and cutoff values are chosen to maximize total diagnostic accuracy (minimize total number of errors).

**Fig. 5:**
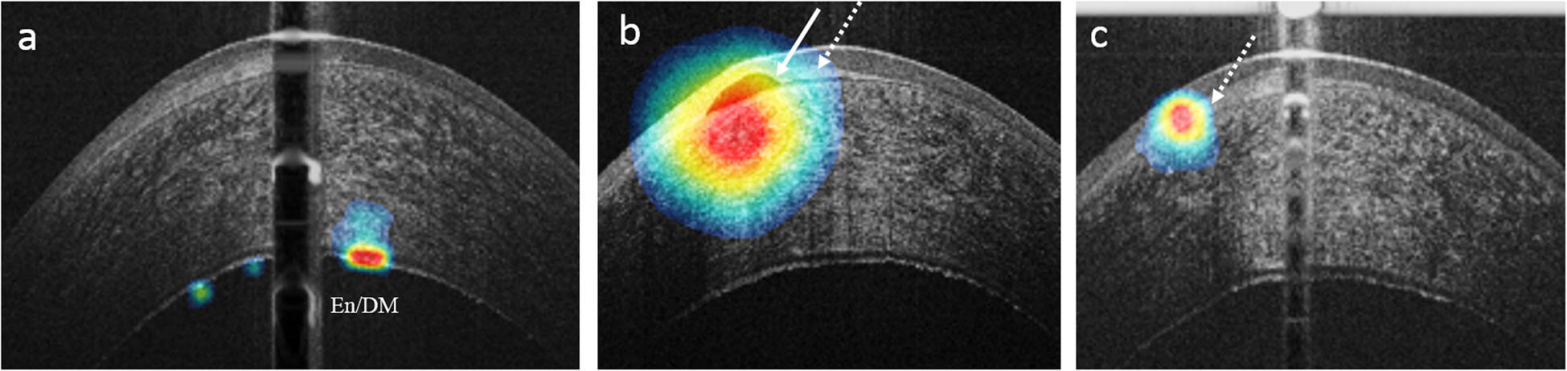
OCT heatmap overlaid on an OCT image, highlighting the learned discriminative features in FECD. These visualizations are generated automatically. The extracted features include the endothelial-Descemet complex (En/DM) in (**a**), subepithelial bullae (white arrow, **b**), and subepithelial scarring (white dashed arrow, **b** and **c**)

Figure 5 shows the visualization of discriminative tomographic features in FECD as learned by the created model, highlighting the pathological changes in the subepithelial region and the posterior part of the cornea including the endothelium-Descemet complex.

## Discussion

FECD is a disease of the corneal endothelium with secondary changes in Descemet’s membrane, stroma, sub-basal nerve plexus and the epithelium [43]. AS-OCT is a proper diagnostic tool to evaluate these changes and might help determine type and timing of intervention, especially as newer therapeutic options enable earlier intervention [9, 44, 45]. This technology has generated large volumes of high-resolution corneal images, with a huge amount of objective data, that make it an excellent target for deep learning modalities [46–49]. However, few studies have focused specifically on automated detection of corneal disease using AS-OCT compared to retinal diseases and glaucoma [32, 50–52]. Using 1172 AS-OCT images, Treder et al. reported a deep learning model to automatically detect graft detachment after Descemet membrane endothelial keratoplasty, and their results showed a sensitivity of 98% with a specificity of 94% [32]. Using OCT images from 12,242 eyes, Yousefi et al. reported a deep learning approach to classify the stages of keratoconus and their results suggest that deep learning can be applied to classifying the status and grading the severity of keratoconic eyes [53]. To the best of our knowledge, our study is the first to use a deep learning approach to diagnose and stage FECD. Regarding this, the aim of our study was to develop and validate a deep learning algorithm to automatically diagnose and grade FECD severity. Our findings showed excellent discrimination between healthy, early-stage FECD and late-stage FECD confirmed by the high accuracy, sensitivity and specificity in diagnosing the 3 classes. Therefore, this approach may be useful to improve the decision-making process for FECD in the context of cataract surgery and corneal transplant.

**Fig. 6:**
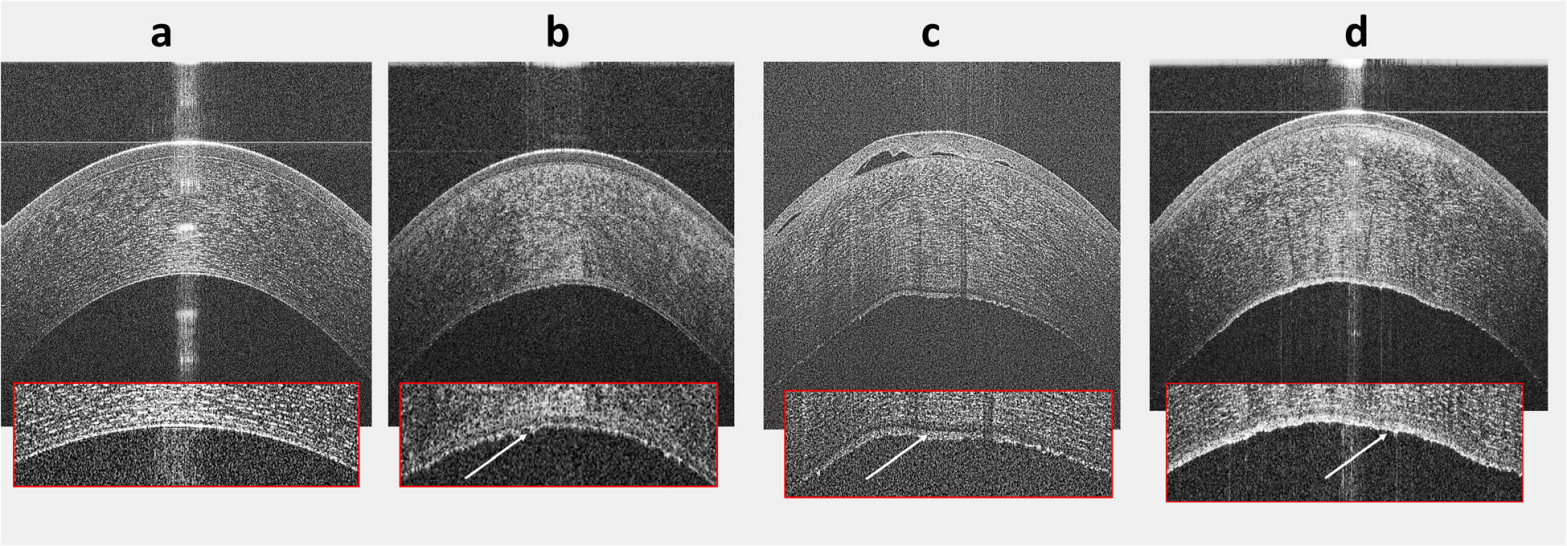
Qualitative discrimination between healthy cornea (**a**), early-stage Fuchs’ endothelial corneal dystrophy (FECD) (**b**), and late-stage FECD (**c**) in AS-OCT images. Healthy and early-stage FECD do not have clinically evident edema on slit-lamp examination (SLE) as shown in the OCT image, yet early FECD demonstrates thickening of the endothelial/Descemet complex (En/DM). Late-stage FECD shows obvious edema with subepithelial bullae. The presets display magnified images of the posterior section of the corresponding cornea. In healthy cornea, the En/DM was visualized as a band formed by 2 smooth regular hyper-reflective lines with a hyporeflective space in between. In FECD, the posterior line had a wavy irregular appearance with areas of focal excrescences representing guttae (white arrows). Figure (**d**) shows a frame from the early stage FECD eye that was misclassified as late-stage FECD. Note the undulations in the endothelial/Descemet complex and a small vesicle underneath the Bowman’s layer

The clinical grading of FECD severity is highly perplexing [16], suggesting that a more objective index of severity is required. The wide range and the diurnal variations in the normal corneal thickness obfuscates its use, especially, to detect subtle corneal edema [16, 17, 54]. Repp et al. reported the relative corneal thickening in FECD as a potential objective parameter to assess the disease severity [16]. However, this can miss a focal paracentral edema, hence, not always effective in evaluating the disease severity. Research points out that mild corneal thickening can exist early in the course of FECD representing subclinical edema, signifying deterioration of endothelial function [54], and can cause a drop in vision [55]. Hence, clinical grading should be interpreted cautiously because this grading might not be the ideal way to evaluate disease severity. It is also possible that corneas with mild edema could be on the brink of requiring a corneal transplant especially after cataract surgery. Krachmer et al. and Louttit et al. described a method to grade FECD using the distribution of guttae and presence of edema [56, 57]. Their grading scales include the existence of edema as a parameter of increased FECD severity but the Krachmer scale states that corneal edema can only be present with extensive guttae. Therefore, the modified scale should recategorize clinically evident edema existing with fewer guttae as lower grade [57]. This confusion may add to the known inter-observer variation of subjective assessments [16] not only for ophthalmologists evaluating the disease in clinical practice, but also for researchers exploring therapies and disease outcomes. In our study, we used a deep learning approach to classify AS-OCT images, and evaluate the diagnostic performance of prediction outputs in each class. The receiver operating characteristics (ROC) analysis demonstrated that its performance was comparable to diagnosis made by cornea specialists (true classification versus the predicted class, Fig. 4). The results of this study highlight the potential utility of deep learning models in identifying early FECD, based on one AS-OCT scan without additional imaging modalities (e.g., Pachymetry, specular microscopy, confocal microscopy) or other information. Furthermore, potential utility was demonstrated for deep-learning-based detection of FECD in 2 different scenarios, that is, from a group of eyes with early stage of disease and from another group of eyes with late stage of the disease. Each of these scenarios might be pertinent in different clinical and research settings. Our recommendation for performing Deep Learning in FECD is to look for early stages without clinically evident corneal edema as this finding can help with optimizing therapeutic decisions. Figure 6 illustrates qualitative features of AS-OCT in FECD and tomographic appearance of the corneal guttae as described elsewhere [22, 23].

To probe the performance of the model, Fig. 5 demonstrates the learned features from AS-OCT images. The highlighted zones in the anterior and posterior parts of the cornea are in agreement with the reported microstructural changes in FECD [22, 23, 58, 59]. Besides, a manual review of the misdiagnosed eye in the early-stage FECD group showed folds in the endothelium/Descemet complex and a small blister underneath the Bowman’s layer (Fig. 6-d). In this eye, 60% of the OCT frames were classified as late-stage, while the rest was classified as early. Also, it’s noteworthy to mention that none of the misclassified images in the late-stage FECD were in the healthy category, while in the early disease, 6% of images were predicted as healthy and 13% was predicted as late FECD. Nevertheless, the overall performance of the model was not affected at both the eye and image levels. This observation agrees with the gradually progressive and asymmetric pathologic nature of the disease. In addition, this could explain the slightly lower performance of the model at the image level compared to per eye level performance.

Our study is not without limitations. First, despite encouraging, the current results stem from a limited number of patients; however, our study demonstrates a substantial, statistically significant ability to discriminate between healthy and FECD eyes (Table 2). Second, the possibility of mild edema cases could be misclassified from early FECD to late stages as this was reliant on clinically evident edema using slit-lamp examination. Interestingly, the slightly higher performance of the algorithm in this study at the eye level could be attributed to the gradually progressive course of FECD. Furthermore, the algorithm in this study was only trained and tested with AS-OCT images captured using the same type of OCT. Therefore, before clinical deployment, as with any novel diagnostic tool, external validation of images from different types of OCT machines should be conducted. Finally, OCT imaging was limited to the central 6 mm of the cornea as the tele-centric probe; there is reduced signal intensity in the peripheral regions [60]. This however, did not negatively impact the quality of the training process as shown by the training and validation curves as well as cross validation.

## Conclusions

This is the first study that presents a deep learning approach to automatically discriminate healthy corneas from early and late FECD disease. Here, we report that our deep learning algorithm can be used as a potential objective diagnostic tool for grading the severity of FECD. The present results may serve as a benchmark for deep-learning-based approach to accurately distinguish normal corneas and cases with guttae but no edema from FECD cases, from the very mild or subclinical edema. Our present work may help stimulate the future development of automated OCT corneal image analysis tools not only in the detection of early FECD but also in monitoring disease progression and customizing therapeutic interventions.

## Data Availability

The datasets used and/or analyzed during the current study are available from the corresponding author on reasonable request.

## Abbreviations

FECD: Fuchs’ endothelial corneal dystrophy
En/DM: Endothelium-Descemet’s complex
HD-OCT: High-definition optical coherence tomography
AS-OCT: Anterior segment optical coherence tomography
ROC: Receiver operating characteristics
AUC: Area under curve
SD: Standard deviation
GEE: Generalized estimating equations
VGG19: Visual geometry group with 19 layers
